# The association of academic tracking to depressive symptoms among adolescents in three Caribbean countries

**DOI:** 10.1186/1753-2000-4-16

**Published:** 2010-05-28

**Authors:** Garth E Lipps, Gillian A Lowe, Sharon Halliday, Amrie Morris-Patterson, Nelson Clarke, Rosemarie N Wilson

**Affiliations:** 1 Department of Sociology, Psychology and Social Work, University of the West Indies - Mona, Kingston, Jamaica; 2 Department of Community Health and Psychiatry, University of the West Indies - Mona, Kingston, Jamaica; 3 Ministry of Health and the Environment, Government of St. Kitts and Nevis, Basseterre, St. Kitts and Nevis; 4 Ministry of Health, Government of St. Vincent and the Grenadines, Kingstown, St. Vincent and the Grenadines; 5 School of Clinical Medicine and Research, The University of the West Indies - Bahamas, Nassau, Bahamas; 6 Sasha Bruce Youthwork Inc., Washington, D.C., USA

## Abstract

**Background:**

Students who are tracked into low performing schools or classrooms that limit their life chances may report increased depressive symptoms. Limited research has been conducted on academic tracking and its association with depressive symptoms among high school students in the Caribbean. This project examines levels of depressive symptoms among tenth grade students tracked within and between high schools in Jamaica, St. Vincent and St. Kitts and Nevis.

**Methods:**

Students enrolled in grade ten of the 2006/2007 academic year in Jamaica, St. Kitts and Nevis and St. Vincent were administered the Beck Depression Inventory II (BDI-II). In Jamaica and St. Vincent, academic tracking was operationalized using data provided by the local Ministries of Education. These Ministries ranked ordered schools according to students' performance on Caribbean school leaving examinations. In St. Kitts and Nevis tracking was operationalized by classroom assignments within schools whereby students were grouped into classrooms according to their levels of academic achievement. Multiple regression analyses were conducted to examine the relationships between academic tracking and BDI-II depression scores.

**Results:**

A wide cross-section of 4^th ^form students in each nation was sampled (n = 1738; 278 from Jamaica, 737 St. Kitts and Nevis, 716 from St. Vincent; 52% females, 46.2% males and 1.8% no gender reported; age 12 to 19 years, mean = 15.4 yrs, sd = .9 yr). Roughly half (53%) of the students reported some symptoms of depression with 19.2% reporting moderate and 10.7% reporting severe symptoms of depression. Students in Jamaica reported significantly higher depression scores than those in either St. Kitts and Nevis or St. Vincent (p < .01). Students assigned to a higher academic track reported significantly lower BDI-II scores than students who were assigned to the lower academic track (p < .01).

**Conclusions:**

There appears to be an association between academic tracking and depressive symptoms that is differentially manifested across the islands of Jamaica, St. Kitts and Nevis and St. Vincent.

## Background

Many young students in the Caribbean face an educational system that places them into secondary schools based upon their performance in critical competency examinations at the end of elementary school. Students who are assigned to lower tracked secondary schools or classrooms may feel their career paths and future are decided for them at the age of ten to twelve. This may be associated with increased feelings of hopelessness and depression. While academic tracking is one factor possibly associated with depressive symptoms, past research has suggested that students who reported high levels of depressive symptoms may be less motivated to achieve academically, have poorer cognitive skills [1] and have lower academic aspirations [2], all of which may lead students to be assigned to lower academic track.

The Caribbean educational system provides an ideal opportunity to examine the relationship between academic tracking and students' emotional health. In this paper we explore the research question, "what association does the highly tracked educational systems in Jamaica, St Vincent and St Kitts and Nevis have with adolescents' depressive symptoms?" While there are many different factors that are associated with depressive symptoms in adolescents, we argue that one factor may be the overt tracking of students into schools and between classrooms such that students assigned to low performing, less academically oriented schools or classrooms may experience higher levels of depressive symptoms.

In the Caribbean, low performing secondary schools are those whose students historically pass fewer critical competency examinations at the end of high school. Many countries endorse and create overt educational tracks whereby the best and brightest students are tracked into the most academically challenging classrooms and schools. Students who attend these "elite" schools often are rewarded with greater social and occupational positions in adulthood[3,4].

### Depression in high school students

Major depression was the fourth most prevalent human disease in 1990 and is expected to rank second by the year 2020 [5] based on research conducted in Europe and North America. It is the most common psychiatric disorder of European and North American adolescents [6]. Adolescent depression in these populations has been linked to psychiatric and substance abuse, unplanned pregnancy, academic and social derailment and most seriously, attempted and completed suicide [7]. Longitudinal studies of depressed European and North American adolescents have documented high rates of recurrence, a progression of the problem into chronicity and conversion into adult affective disorders [7]. Epidemiological studies of these populations suggest that female adolescents are at greater risk for developing depression, with this difference first emerging sometime around the period of mid adolescence [8]. Past research using Caribbean samples has found high levels of moderate to severe depressive symptoms among adolescents. These range from to 24.5% in St. Kitts & Nevis [9], 25.3% in Trinidad [10] to 40.6% in Jamaica [11]. These studies suggest that Caribbean adolescents tend to report moderate to severe levels of depressive symptoms at a higher rate than those reported in studies using North American and European samples [12-16].

While much research has been conducted using North American and European samples of adolescents [12-15], relatively little research has examined depressive symptoms among adolescents in the Caribbean [9,11,17,18]. In contrast to North American and European societies, several factors in Caribbean society may place students at an elevated risk for experiencing depressive symptoms, including high levels of general poverty [19], high prevalence of female headed single parent families [20], and lower levels of general education [21]. The combination of these social and economic conditions may combine to create a depressogenic environment. Living under these conditions may lead youth to experience a sense of futility and hopelessness which contributes to the development of depressive symptoms.

Compounding these social, economic and personal conditions, the school systems of various Caribbean islands engage in educational practices that contribute to the social and economic differences between their citizens [3,4]. Many countries endorse and create overt educational tracks whereby the best and brightest students are tracked into the most academically challenging classrooms and schools. Students who attend these "elite" schools often are rewarded with greater social and occupational positions in adulthood [4].

While the islands of the Caribbean share a common past and many social and economic similarities, their educational systems are distinct. In particular, each nation has chosen a slightly different approach to the educational tracking of students. The three nations that are the focus of this research have developed three different approaches to the tracking of students.

### The Jamaican educational context

The Jamaican system of secondary education is highly stratified with schools divided into traditional high schools and upgraded secondary schools, paralleling Jamaica's socio-economic class differences [4,22]. Strudwick [4] in his analysis of Jamaican secondary schools noted that few students from the lower social classes (less than 4%) attended a traditional high school, while approximately 75% of students attending traditional high schools were from the upper or middle class backgrounds. Similarly, only 33% of students attending upgraded secondary schools were from upper or middle class backgrounds.

Interestingly, while students are assigned either to a coveted traditional high school or a less prized upgraded secondary school based on their scores on an exit examination administered at the end of elementary school - The Grade Six Achievement Test (GSAT), this system is not a pure meritocratic system as students academic achievement is highly correlated with their social class [4]. Only students who have exceptionally high scores on the exit examination are selected for attendance in traditional high schools [23].

This assignment to schools has practical consequences for students' future opportunities. Attendance in high academic performing traditional high schools increases students' chances of obtaining high grades (e.g. A grades) on the critical competency examinations given at the end of high school (grade eleven)[4]. High grades in these exams are needed to move on to university and technical colleges which are the pathways to higher paying and more prestigious occupations [3,4]. Consequently, students who are placed in upgraded secondary schools may be at higher risk for the development of depressive symptoms.

### The educational context in St. Vincent

Similar to the Jamaican educational system, students in St. Vincent are assigned to either a traditional or upgraded secondary school based on their performance on a high school placement examination. The five hundred students with the highest test scores are given the opportunity to choose a traditional high school of their choice. All other students who score below the top 500 are placed by the Ministry of Education into a lower academically performing non-traditional government high school close to their place of residence [24].

Students who attend traditional high schools are more likely to score highly in the regional critical competency, exit examinations. In 2008 the traditional high schools had pass rates ranging from 73% to 93% while the non-traditional high schools received pass rates ranging from 37% to 60% [24]. Obtaining passes in several courses is a requirement for further education as well as securing a good job.

### The educational context of high schools in St. Kitts and Nevis

Like the Jamaican and Vincentian educational systems, the school system in St Kitts and Nevis has an examination for students leaving their elementary schools and entering high schools - The Grade Six Test of Standards (GSTS) [25-27]. However, unlike Jamaica and St. Vincent this test is not used to track students into different high schools, as students from four different elementary schools feed into a single high school nearest to where they live. As such, students are tracked to classrooms within high schools rather than to high or low achieving high schools. The GSTS along with students' classroom grades in grades five and six are used to assign students into four to six academic tracks within each high school - six tracks in the larger high schools and four tracks in the smaller high schools [25]. Where students' school grades are consistent with their performance on the GSTS, the GSTS is used to assign students to a particular classroom track within their local high school. However, when students' classroom grades in grades five and six suggest they should be placed in a higher track than that suggested by the GSTS, then classroom grades are used to assign students to an academic track within their local high school [25].

### The impact of academic tracking on students' achievement and adjustment

Research has recently begun to examine the relationships between academic tracking and students' academic and socio-emotional adjustment. In terms of academic achievement, there have been conflicting findings. Some studies have suggested that academic tracking may lead to increases in students' academic achievement [28,29] while other research suggests that this may be true only for high achieving students [30,31]. Other research has focused on the association of academic tracking to student misconduct and school violence [32,33]. This research suggests that there may be an association between assignment to a low academic track and higher levels of self-reported reported misconduct [32,33]. Similarly, societies which track students into high achieving or low achieving schools or classrooms have higher levels of school violence then societies which do not track students [32].

Research has examined the effect that tracking has on students' later occupational, economic and human capital. Using data from several large, international, and nationally representative surveys of European, North American, and Asian countries, Brunello and Checchi [34] found that being assigned to a low academic track reduced students' chances of being employed, their wages when employed, their occupational attainment, the probability of attendance in post-secondary education and their highest level of education [34]. Further, the longer time students were subjected to tracking the greater the negative effects for low tracked students [34]. Educational tracking was also found to combine with parental background compounding the negative effects of tracking [4,34]. Within the Caribbean, research has suggested academic tracking to high schools is positively associated with students' later level of education, income and occupation [4].

Researchers in Great Britain [35] have studied the impact that educational setting (the rigid grouping of students to homogenous ability groups within schools) has on students' psychological and future occupational success. Boaler [35] found that rigid academic tracking had a strong, negative psychological effect on students' attitudes towards school, life and their future opportunities [35]. Students who were assigned to all but the top ability group reported they felt that their class assignment had constrained their future academic and occupational achievements as well as setting them up for low attainment in life [35]. Students assigned to low tracks reported feeling as if they had been placed in a psychological prison that limited their knowledge of their capabilities [35]. Further, the assignment to a lower academic track broke their ambition. In following up students five years after graduation, students who had attended schools that did not rigidly track students by ability levels attained significantly more prestigious jobs [35].

### Rationale and hypotheses

There has been a paucity of research on depressive symptoms among Caribbean adolescents and even less on the association of academic tracking with adolescent students' emotional health in the Caribbean. The present study sampled students from various academic tracks in high schools in three Caribbean nations using a cross-sectional research design. We hypothesised that students who attended lower academic tracks in high schools would have higher levels of depressive symptoms than those in higher academic tracks. Consistent with past research, we further hypothesized that girls will report higher symptoms of depression.

## Methods

### Sample

Grade ten students from Jamaica, St. Kitts and Nevis and St. Vincent were chosen to take part in the research project. Students attending the tenth grade were chosen as they have been exposed to the system of academic tracking for several years, were not expected to sit critical competency examinations in the current academic year and had made the transition to secondary education. Additionally, research has demonstrated that students in the lower grades were more likely to experience emotional problems [36]. No participants refused to take part in the study. Participants expressed strong interest in the study and were therefore highly motivated to take in the research. However, youth who were not present on the day of data collection were not included in the sample of each country. Table 1 displays the demographic features of students by gender, maternal education, age and academic track for each country.

**Table 1:** Demographic features of sampled students

	Jamaica		St Kitts		St Vincent	
	N	%	N	%	N	%
Gender						
Male	115	41.4	371	50.3	384	53.6
Female	145	52.2	352	47.8	382	46.4
Missing	18	6.5	14	1.9	0	0.0
Maternal Education						
Secondary or Less	130	46.8	437	59.3	487	68.0
Post-Secondary	148	53.2	204	27.7	177	24.7
Missing	0	0.0	96	13.0	52	7.3
Age						
14	40	14.4	72	9.8	65	9.1
15	179	64.4	309	41.9	342	47.8
16	59	21.2	279	37.9	191	26.7
17	0	0.0	77	10.4	88	12.3
18	0	0.0	0	0.0	29	4.1
Academic Track						
Low	115	41.4	189	25.6	360	50.3
High	163	58.6	548	74.4	356	49.7

#### Jamaica

A cross-section of grade ten students from traditional and non-traditional high schools in urban and rural Jamaica was sampled (n = 278 students; 41% males, 52% females and 7% not stated; age 14 to 16 years, mean = 15.0 yrs ± 0.6 yr: Table 1). Schools were selected for participation based on their level of academic performance and the category of school. This selection was done by the researchers using published data of academic achievement by schools of students who sat the mandatory Caribbean Secondary Education Exams [37]. One-hundred and sixty three students were recruited from traditional high schools while 115 students were recruited from non-traditional high schools (Table 1). The schools sampled included a traditional urban high school, a non-traditional urban high school, a rural traditional high school and a non-traditional rural high school. Classes within schools were selected so as to have a balance between high and low performing students. Traditional and non-traditional schools differed by gender (chi-square [2] = 18.2, p < .05), age (chi-square [2] = 6.0, p < .05), and maternal education (chi-square [1] = 19.8, p < .05) such that traditional high schools tended to have a higher percentage of females, younger students, and more highly educated mothers (Tables 1 and 2).

#### St. Vincent

Data were collected from 716 grade ten students attending eight secondary schools across the island of St. Vincent (Table 1). Schools were selected for participation based on their level of academic performance and the category of school (traditional versus non-traditional high school). A local researcher selected the schools based on discussions with officials of the Ministry of Education of St. Vincent regarding the specific types of schools required for the research sample. The Ministry suggested non-traditional and traditional high schools to be sampled. Schools were selected such that three traditional and five non-traditional schools were sampled. Of the eight schools sampled, two were boys' schools, two were girls' schools and the remaining four were co-educational schools. Students within each school were randomly sampled to take part in the study. Of the 716 participants sampled, 384 were female and 332 were male (Table 2). Students ranged in age between 12 and 19 years (mean = 15.5 years, sd = 1.0).

**Table 2:** Gender by type of school

Jamaica				
		Non-Traditional	Traditional	Total
Female	n	53	92	145
	%	20.4%	35.4%	55.8%
Male	n	46	69	115
	%	17.7%	26.5%	44.2%
St. Vincent				
		Non-Traditional	Traditional	Total
Female	n	199	185	384
	%	27.8%	25.8%	53.6%
Male	n	161	171	332
	%	22.5%	23.9%	46.4%
St. Kitts and Nevis				
		Low Track	High Track	Total
Female	n	94	277	371
	%	13.0%	38.3%	51.3%
Male	n	90	262	352
	%	12.4%	36.2%	48.7%

#### St. Kitts and Nevis

The researchers contacted officials within the Ministry of Education regarding the selection of schools. Because of the high interest in the findings of the study, the Ministry of Education requested that all schools in St. Kitts and Nevis be sampled. As such, a near census of grade ten students attending all high schools in Saint Christopher (St. Kitts) and Nevis were surveyed (n = 744; Table 1). A list of all schools providing secondary education in St. Kitts and Nevis was obtained from the Ministry of Education. Schools were visited and all grade ten students in attendance on that day were surveyed. The sample consisted of nearly equal percentages of female (50.4%) and male (47.6%) students with 2% the sample not reporting their gender. Students in our sample ranged from 13 to 19 years of age (mean = 15.5 yrs ± 0.8 yr: Tables 1 and 2).

## Measures

### Beck Depression Inventory - II (BDI-II)

The Beck Depression Inventory (BDI-II) [38] is a 21 item questionnaire which examines the cognitive, behavioural, affective and somatic symptoms of depression. Each item of the BDI-II is comprised of a series of rank ordered statements. Each statement is assigned a score from 0 to 3 reflecting the severity of the symptom. Students were asked to circle the number associated with the statement that most accurately describes their feelings during the past two weeks. Previous research suggests that the BDI-II is reliable in North American samples of adults [38]. Studies using both non-clinical [39,40] and clinical samples of adolescents [41] have reported acceptable internal consistency reliabilities, with coefficient alphas ranging from .87 to .94. Within a sample of Jamaican adolescents the BDI-II had a high internal consistency reliability (α = .87; [42]). In the current sample the BDI-II was found to have a high internal consistency reliability (α = .88). Past research suggests that the BDI-II is valid across different cultures [15,38], even in cultures that place a high stigma on psychological problems [43]. Based on their BDI-II scores, adolescents in this study were divided into minimal (13 or less), mild (14 to 19), moderate (20 to 28) or severe (29 or higher) symptoms of depression. While depression is a clinical diagnosis requiring professional assessment of adolescents by a clinical psychologist or psychiatrist, the BDI-II simply provides information on levels of depressive symptoms. Some youth, but not all, who have high BDI-II scores may be judged by clinicians to have major depressive disorder.

### Demographic data

A variety of information on students' demographic features, including their age and gender, was collected using a series of brief questions. Students were asked to report their exact age in years on their last birthday and their gender. Students were also asked to report on their mothers' highest level of education using the categories of no schooling, kindergarten, primary, secondary/high school, trade/vocational, associate degree, bachelor's degree and graduate degree. For the analyses these categories were collapsed into secondary education or below or post-secondary education.

### Procedure

Prior to the start of the project, a research assistant liaised with the Ministry of Education in each country and the schools selected to participate in the project in order to describe the study's aims and obtain consent for conducting the project. Students in the classrooms selected for this project were given an informed consent form for their parents to complete. Data for the project were collected from students during one of their regularly scheduled classes. All students in a classroom whose parents provided their informed consent for their adolescent to take part in the project were given a package of instruments to complete. This package consisted of an informed consent form for the adolescents, the BDI-II, and a series of questions regarding their demographic features.

### Statistical Analyses

Several statistical analyses were conducted. Preliminary analyses were conducted to check and correct data capture problems using simple frequency and cross-tabulations. A check was made on the extent of missing values for each question on the questionnaire. No question was found to be missing more than 10% of responses. On this basis the mean score was substituted for missing values on each question. Basic descriptive statistics were then run to check that the data met the statistical assumptions required by multiple regression analysis. These checks found that the data met the assumptions needed to perform multiple regression. As such, no transformations were made to the data. Multiple regression analyses were conducted to examine the simple associations of age and gender, maternal education, academic track and country with BDI-II depression scores, as well as the interactive association of country with gender, maternal education and academic track. In this regression analysis, BDI-II depression scores served as the dependent variable while age, gender, maternal education, academic track, country and the interactions of country with gender, maternal education and academic track served as the independent variables. Dummy coded predictors were used to represent gender (0 = Female, 1 = Male), maternal education (0 = Secondary education or less, 1 = Post-Secondary education), and academic track (0 = Low Track, 1 = High Track) in the regression analyses. The category of low track includes students who are attending schools or classroom associated with low academic achievement, while the category of high track includes students who are attending schools or classroom associated with high academic achievement.

## Results

### Prevalence of Depression

Simple frequency analyses were conducted to examine the extent of depressive symptoms across the schools in this study. Nearly half (53%) of all students reported mild to severe symptoms of depression with 19.2% reporting moderate and 10.7% reporting severe symptoms of depression. This patterning of depression scores however, was not uniform across countries (Table 3). Nearly two-thirds (64%) of all adolescents in Jamaica reported mild to severe symptoms of depression with 26.3% reporting moderate symptoms and 14.4% reporting severe symptoms of depression. St. Kitts and Nevis had the lowest levels of depressive symptoms with only 46.3% of students reporting mild to severe symptoms of depression, 14.0% of students reporting moderate symptoms and 10.7% reporting severe symptoms of depression. St Vincent fell in between Jamaica and St Kitts and Nevis with 55.4% of students reporting mild to severe symptoms of depression, 21.8% reporting moderate symptoms and 9.2% reporting severe symptoms of depression. Tracking appeared to be differentially associated with level of depressive symptoms by country (Table 4) such that higher levels of depressive symptoms were reported by students in the low academic track in Jamaica.

**Table 3:** Depressive symptoms by country

	Minimal	Mild	Moderate	Severe
Jamaica	36.0%	23.4%	26.3%	14.4%
St. Vincent	44.6%	24.4%	21.8%	9.2%
St. Kitts and Nevis	53.7%	21.6%	14.0%	10.7%

**Table 4:** Depressive symptoms by country and by academic track

	Track	Minimal	Mild	Moderate	Severe
Jamaica	Low	21.7%	27.8%	31.3%	19.2%
	High	46.0%	20.3%	22.7%	11.0%
St. Vincent	Low	40.3%	25.8%	22.5%	11.4%
	High	48.9%	23.0%	21.1%	7.0%
St. Kitts and Nevis	Low	46.0%	23.8%	18.0%	12.2%
	High	56.4%	20.8%	12.6%	10.2%

### Regression analyses

To explore the relationship of gender, country, maternal education and academic tracking a hierarchical multiple regression analysis was conducted (Table 5). The regression analysis used mothers' highest level of education, gender, country, and academic tracking as independent variables in the analyses and BDI-II scores as the dependent variable. In the first stage of the analysis mothers' highest level of education, gender, country, and academic tracking were entered simultaneously into the regression equation. The second stage of analysis entered the interactive effects of country by gender, country by maternal education, country by academic track. Results of this analysis indicated that there were significant differences in BDI-II depression scores by country, academic tracking, gender, and maternal education.

**Table 5:** Results of regression analyses of school related factors predicting BDI-II depression scores, controlling for gender and maternal education

Predictor	B	Beta	t	Chg R^2^
Stage One - Main Effects				
Jamaica	2.57	.10	3.52*	
St. Vincent	.50	.02	.90	
Academic Track	-2.44	-.12	-4.65*	
Gender	-4.24	-.21	-8.54*	
Mother's Education	-1.61	-.08	-2.98*	
				
Stage Two - Interaction Effects				
Jamaica by Academic Track	2.68	-.08	-1.76	.003
St. Vincent by Academic Track	.72	.03	.62	
Jamaica by Gender	4.73	.12	2.30*	.008*
St. Vincent by Gender	-.11	-.00	-.10	
Jamaica by Mother's Education	1.88	.05	1.28	.001
St. Vincent by Mother's Education	.52	.02	.44	

* p < .05

Jamaican students reported significantly higher BDI-II scores (t_[1556] _= 3.52, p < .01; Table 5) than students attending high schools in St. Kitts and Nevis. On average students in Jamaica reported BDI-II depression scores that were 2.5 points higher than students in St. Kitts and Nevis. In contrast, St. Vincent students did not report significantly higher BDI-II depression scores than students in St. Kitts and Nevis (t_[1556] _= .90, p > .05; Table 5).

Students assigned to a higher academic track reported significantly lower BDI-II scores than students who were assigned to the lower academic track (t_[1556] _= -4.65, p < .01; Table 5). On average, students in the higher academic track scored 2.4 points lower in the BDI-II depression scale than those assigned to the lower track.

Gender also made a unique contribution to the prediction of students' depression scores. Male students scored 4.2 points lower than their female counterparts on the BDI-II inventory (t_[1556] _= -8.54, p < .01; Table 5).

Maternal education was used as a proxy for social class as a large number of Caribbean families are headed by single women [43,44]. On average, students whose mothers had a post-secondary education scored 1.6 points lower on the BDI-II scale (t_[1556] _= -2.98, p < .01; Table 5) than students whose mothers had graduated from or not completed secondary school.

Only one of the interactive effects was statistically significant. The country by gender interaction was statistically significant (R^2 ^chg = .008, F(2, 1554) = 6.50, p < .01) such that male students in Jamaica reported significantly higher BDI-II scores than their counterparts in the other two islands (t_[1556] _= 3.30, p < .01; Table 5). The interaction of country by academic tracking approached statistical significance (R^2 ^chg = .003, F(2, 1554) = 2.75, p = .06). There was no statistically significant interaction of country by maternal education (R^2 ^chg = .001, F(2, 1554) = .08, p > .05).

## Discussion

Based on the findings of the present study, a profile of a student at risk for high levels of depressive symptoms in the Caribbean can be created. This student would most likely be a Jamaican female who was placed in a low academic track and whose mother did not attain a post-secondary education.

### The interaction of gender and country

Consistent with international findings, female students across the three islands reported significantly higher depressive symptoms than males. Some possible reasons for this gender difference include physiological factors such as hormonal changes [8], body image issues [45], and a more negative attributional style [46]. Additionally, consistent with international research [47] it is possible that gender role expectations and family structure may play a role. In the Caribbean, the majority of households are headed by single females. As such, the role of care giving in the family may fall to the oldest female child, placing added stress on her leading to increased risk for depression. This role may extend into adulthood limiting girls' hopes for future education and occupational success. The school system may also play a role in differences in levels of depressive symptoms by gender. Research by West and Sweeting [47,48] has suggested that the educational system may place higher academic expectations on female students thereby increasing their propensity to experience depressive symptoms.

In St. Kitts and Nevis and St. Vincent female students reported substantially higher levels of depressive symptoms than their male counterparts. However, with respect to the Jamaican sample, male and female students reported similar, high levels of depressive symptoms. The double educational tracking experienced in Jamaica may create a depressogenic environment for both genders hence negating the gender difference in depressive symptoms which is usually seen. Supporting this interpretation, previous research using Jamaican high school students [18] reported that adolescents from the higher social classes placed in a high achieving traditional high school manifested gender differences in depressive symptoms which have been found in past international research. In addition, some research has suggested that male youth in Jamaica may receive very little social and emotional support from significant adults. A qualitative study done by Evans [22] noted that male students are often treated worse than female students by their teachers. Research by UNICEF [44] has also found that many Jamaican mothers discriminated against their sons. In combination, the lack of support in both the home and school environments may lead male students to experience higher levels of depressive symptoms.

### Differences in depressive symptoms by academic track

Past research has found that academic tracking is associated with depressive symptoms [49] such that students who were assigned to lower academic sets (tracks) reported higher levels of depressive symptoms. In this study, students tracked to lower achieving schools and classrooms reported significantly higher levels of depressive symptoms. Consistent with research in England [35], being tracked to a lower achieving school or classroom may be associated with a sense of hopelessness and despair leading students to express higher depressive symptoms. Research by Boaler [35] on English school leavers has suggested that children assigned to high academic tracks were more likely to secure more middle class occupations in later life than those assigned to lower tracks who were more likely to obtain working class jobs. Similarly in the Caribbean, students assigned to higher tracks are more likely attend college or university and obtain higher paying professional and managerial positions [3].

Academic tracking however may not be the sole possible explanation for the study's findings. Several other factors may be associated with the types of schools adolescents' attended. These include the intergenerational transmission of depression from mothers to children [16], community factors such as violence, social disorganization, crowding, and lack of social amenities such as adequate roadways, and recreational and educational facilities [50].

### Differences in depression by country

Of the three islands Jamaican students reported the highest depressive symptoms. One possible explanation for why students in Jamaica report higher levels of depressive symptoms is that Jamaican students experience double tracking - tracking between schools and between classrooms within schools. In general, Jamaican students attend either a higher achieving traditional high school or a lower performing upgraded secondary school. Attendance in a traditional high school provides many opportunities for social advancement via better paying jobs, and increased opportunities for post-secondary education. In contrast, students who attend upgraded secondary schools have fewer such opportunities [3]. Within schools, students are further tracked into higher or lower performing classrooms. Both of these factors may contribute to higher levels of depressive symptoms among Jamaican students.

In St Kitts and Nevis the educational programme tracks students into classrooms within schools providing a more heterogeneous school environment, while the educational system of St. Vincent tracks students into schools but not within classes in the schools. Past research has shown that overt tracking of students into classrooms and schools is associated with higher depressive symptoms [11].

Differences in neighbourhood structure may provide an additional explanation for the higher levels of depressive symptoms in Jamaican students. Geographically, Jamaica has clearly demarcated areas which are stereotyped and stigmatized as being crime and violence prone, low income, inner-city areas [51]. Many of these communities are quite homogeneous contributing to the social toxicity of the environment. Higher levels of social toxicity have been linked to students' depressive symptoms[52].

Differences between neighbourhoods are less pronounced in St Kitts and Nevis as well as St. Vincent. Communities in St. Kitts and Nevis and St. Vincent have a greater mixture of income levels, educational levels, and are more socially integrated with the surrounding wealthier communities. In addition, the differences in housing and amenities are more evenly distributed in St. Kitts and Nevis and St. Vincent across communities and social classes. This greater diversity in communities may explain the lower levels of depressive symptoms among students in St. Kitts and Nevis as well as St. Vincent.

### Maternal education and depression

Students whose mothers had a post-secondary education reported significantly lower symptoms of depression. Parental education often opens social and educational opportunities for children [53,54]. Consequently, students whose mothers have not achieved a post-secondary education may have fewer opportunities and resources for upward mobility in the Caribbean as they are less able to garner the resources and contacts needed. This differential access to resources may be associated with a sense of hopelessness and despair leading students to express higher depressive symptoms. In contrast, well educated mothers may serve as role models for female students, thereby providing them with increased hope and motivation to succeed.

Of interest, some research [55] has found that the burden of childcare may shift to adolescent girls whose mothers who have higher levels of education and are more likely to be employed. On the other hand, mothers with less than a post-secondary education may need to supplicant their income with additional employment, hence further decreasing time spent with their children, and placing increased burdens for care-giving on their adolescent female children. This is particularly relevant in the Caribbean context where most households are headed by single women. Regardless of the mechanism, adolescent girls may be overwhelmed by the responsibilities they are forced to take on, resulting in increased depressive symptoms.

### Extension of the findings to other social and cultural contexts

The results of this study may be applicable to other countries where the educational systems are stratified and divided along social and academic dimensions. Countries such as Scotland and England, which have a similar educational system to those in Jamaica and St. Vincent may have also place their students at an elevated risk for depressive symptoms.

### Limitations of the research

The findings of this research project have several limitations. First, it is possible that children who did not want to take part in the study did not attend school on the day of data collection. Consequently, this may have influenced the findings. However, many of the participants reported that they wanted to take part in the study and expressed to the researchers a strong interest in the findings. The opinions of many of the participants had never been explored in research and the topic was of great interest and concern to them. As such, many participants were keen to take part in the research.

## Conclusions

There appears to be an association between academic tracking and depressive symptoms that is differentially manifested across the islands of Jamaica, St. Vincent and St. Kitts and Nevis. In addition, gender appears to be associated with depressive symptoms in each of the three Caribbean islands.

## Competing interests

The authors declare that they have no competing interests.

## Authors' contributions

All authors of the paper have seen, reviewed and approved this manuscript. GAL and GEL were involved in all aspects of this paper. GEL and GAL shared in the conception and planning of the research project, conducted the statistical analyses and interpreted the findings. GEL wrote the method and results sections for this paper, GAL composed the introduction and GEL and GAL collaborated on the discussion section of the paper. SH, AM, NC and RNW assisted with the conceptualization of the paper, coordination and data collection as well as the review of draft versions of the manuscripts.
